# Exosomes derived from human umbilical cord mesenchymal stem cells reduce tendon injuries via the miR-27b-3p/ARHGAP5/RhoA signaling pathway: Influences of hucMSC-exosomes in tendon injuries

**DOI:** 10.3724/abbs.2021026

**Published:** 2022-01-26

**Authors:** Qinghui Han, Shuguang Wang, Dawei Chen, Di Gan, Tao Wang

**Affiliations:** Department of Orthopaedics and Traumatology Shanghai East Hospital School of Medicine Tongji University Shanghai 200120 China

**Keywords:** exosomes, RhoA, ARHGAP5, miR-27b-3p, human umbilical cord mesenchymal stem cells

## Abstract

Tendon injuries are common clinical issues resulted from tissue overuse and age-related degeneration. Previous sutdies have suggested that exosomes secreted by mesenchymal stem cells (MSCs) contribute to tissue injury repair. Here, we provide evidence for a critical role of human umbilical cord mesenchymal stem cell (hucMSC)-derived exosomes in reducing tendon injury by activating the RhoA signaling. Treatment of primary injured tenocytes with hucMSC exosomes increases cell proliferation and invasion, which correlates with increased RhoA activity. RhoA mediates the effects of hucMSC exosomes, as treatment of primary injured tenocytes with the RhoA inhibitor, CCG-1423, abolishes the effects of hucMSC exosomes on cell proliferation and invasion. Mechanistically, we observe that hucMSC exosomes induce the expression of a microRNA, miR-27b-3p, which targets and suppresses ARHGAP5, a negative regulator of RhoA. Consistent with this observation, ARHGAP5 overexpression suppresses the effects of hucMSC exosomes on cell proliferation and invasion, while knockdown of ARHGAP5 rescues these effects. Finally, we demonstrate the functional significance of our findings *in vivo* using an Achilles tendon injury model and show that treatment with exosomes reduces tendon injury in rats, which correlates with increased RhoA activity and reduced ARHGAP5 expression. Taken together, our findings highlight a critical role of hucMSC exosomes in reducing tendon injury via miR-27b-3p-mediated suppression of ARHGAP5, resulting in RhoA activation, and leading to increased cell proliferation and invasion of primary injured tenocytes.

## Introduction

Tendon injuries are the most frequent soft tissue injuries resulted from tissue overuse and age-related degeneration [ 1– 3]. As many as 50% of sports-related injuries involve tendons [4] and a considerable proportion of the elderly populations are prone to tendon injuries due to aging [1], resulting in significant morbidity and healthcare costs. Tendon tissues have limited healing capacity [5], highlighting the importance of developing therapeutic approaches that can promote tendon healing and regeneration.

Tendon healing is a gradual process that often results in inferior fibrotic tissue formation, which increases the risk of re-injury [ 6, 7]. Due to hypocellularity and hypovascularity of tendon tissues, tendon healing capacity is limited even after surgical repair [5]. Often, the healed tendon has inferior mechanical properties, resulting in long-term pain, discomfort, and even disability in many patients [2]. The utility and regenerative potential of mesenchymal stem cells (MSCs) in tissue engineering have long been recognized, with several studies demonstrating the improvements in tendon regeneration using MSC-based strategies [ 8– 10]. In response to stimuli, MSCs can differentiate into multiple lineages, including tendon cells (tenocytes) [11]. Human umbilical cord MSCs (hucMSCs) are a source of MSCs and provide many advantages for studying regenerative injury, including faster renewal properties and painless collection procedures [12]. Additionally, hucMSCs potentially promote tendon healing and regeneration [13].

Exosomes are nanometer-sized vesicles released by almost all cell types in the body including stem cells, and are potent drivers of tissue regeneration [14]. They mediate cell-to-cell communications by delivering lipid, protein, and nucleic acid cargos [15]. Exosomes function as important components of MSCs and play significant roles in tissue damage repair. MSC-derived exosomes are increasingly recognized as new biological pro-regenerative therapeutic agents for different types of tissue injuries, and have been used in myocardial infarction, stroke, kidney injury, and osteochondral injury settings [ 16, 17].

The Rho family of small GTPases orchestrates several important biological processes, including cell cycle progression and actin cytoskeleton dynamics, and their aberrant signaling has been linked to human diseases. The majority of Rho family members undergo conformational switching between two states: GTP-bound active and GDP-bound inactive [18]. This switch is tightly regulated by guanine nucleotide exchange factors (GEFs) and GTPase activating proteins (GAPs) [19]. RhoA is a member of the Rho family of GTPases with roles in the regulation of the actin cytoskeleton, which is an important component of tissue repair. It has been reported that RhoA promotes proliferation and migration of injured tenocytes [13], suggesting a potential role in tendon healing. However, how hucMSC exosomes and RhoA signaling in tendon injury repair are related and/or coordinated remain poorly understood.

In this study, we uncovered a crucial role of hucMSC-derived exosomes in reducing tendon injuries via activation of RhoA signaling, resulting in increased cell proliferation and invasion of primary injured tenocytes. RhoA activity was stimulated by exosomes via the miR-27b-3p-mediated targeting of ARHGAP5 which acts a negative regulator of RhoA. Importantly, suppression of RhoA activity using the RhoA inhibitor, CCG-1423 or by ARHGAP5 overexpression abolished the effects of hucMSC exosomes on cell proliferation and invasion. We highlighted the biological significance of our findings by demonstrating that hucMSC exosomes reduced tendon injury in an *in vivo* rat model. Thus, we provide evidence for the therapeutic potential of hucMSC exosomes in tendon healing and preventing re-injury.

## Materials and Methods

### Cell culture

Tendon tissue biopsies were collected as previously described [20] from midportion Achilles tendons of volunteer healthy donors ( *n*=8) with no history of Achilles tendon pain and no structural changes as demonstrated by Color Doppler ultrasound examination, and of patients with Achilles tendon rupture ( *n*=12) at Shanghai East Hospital (Shanghai, China). This study was approved by the Institute’s Ethics Committee. Written informed consent forms were signed by all participants. Tenocytes were maintained in Dulbecco’s modified Eagle medium (DMEM; Hyclone, Logan, USA) containing 10% fetal bovine serum (FBS; Gibco, Paisley, UK) and 1% penicillin-streptomycin (Hyclone) in a humidified incubator at 37°C with 5% CO _2_. HucMSCs at passage 2 were kindly provided by Cyagen Biotechnology Inc (Guangzhou, China), and were maintained in MSC NutriStem® XF medium (Biological Industries, Beit HaEmek, Israel) containing 15% FBS at 37°C in 5% CO _2_, with culture medium refreshed every 2–3 d. HucMSCs at passage 3 were used for subsequent experiments. The immunophenotype of culture-expanded hucMSCs was characterized by flow cytometry based on previously described methods [21]. All hucMSCs showed positive expression for CD90 and CD105 and negative expression for CD11b and CD45.

### Cell transfection

Three small interfering RNAs (siRNAs) targeting human *ARHGAP5* were used for transfection: siARHGAP5#1 (point 1653–1672), 5′-GGAAGUUAUGUUGCUUUGTT-3′; siARHGAP5#2 (point 3531–3550), 5′-GCCUUUGGCACAUCCUGAATT-3′; and siARHGAP5#3 (point 4761–4780), 5′-GCUGAUACAACCACAAUUATT-3′. Injured tenocytes were plated in six-well plates (5×10 ^3^ cells/well) and transfected with siARHGAP5 or negative control siRNA (siNC; 5′-UUCUCCGAACGUGUCACGUTT-3′). To overexpress ARHGAP5, its coding sequence was cloned into the pcDNA3.1(+) plasmid (Addgene, Watertown, USA). Transfection was performed using Lipofectamine 2000 reagent (Invitrogen, Carlsbad, USA) following the manufacturer’s instructions. Then, 6–8 h after transfection, injured tenocytes were replenished with fresh complete medium and cultured for 24 h. The miR-27b-3p mimic 5′-UUCACAGUGGCUAAGUUCUGC-3′, miR-27b-3p inhibitor 5′-GCAGAACUUAGCCACUGUGAA-3′, and negative control (NC) 5′-CAGUACUUUUGUGUAGUACAA-3′ were generated by Genepharm Technologies (Shanghai, China). Transfections were conducted as described.

### Exosome studies

HucMSCs were grown to ~90% confluence and provided with freshly prepared complete medium supplemented with exosome-free FBS for 48 h. The medium was then collected and exosomes were harvested by differential centrifugation based on previously described methods [22] with some modifications. Briefly, supernatants were centrifuged at 2000 *g* for 10 min and then 10,000 *g* for 30 min to remove debris and apoptotic bodies. Subsequently, supernatants were centrifuged at 100,000 *g* for 90 min, followed by washing with phosphate buffered saline (PBS) and purification by centrifugation at 100,000 *g* for 90 min. All centrifugation steps were performed at 4°C. The pellet was resuspended in PBS and sterilized by filtration through a 0.22‐μm filter (Millipore, Billerica, USA). Finally, purified exosomes were resuspended in PBS for the detection of concentration and size distribution using the Flow Nano Analyzer (NanoFCM Co., Ltd, Xiamen, China) according to manufacturer’ s protocol, and the data was processed using NanoFCM software (NanoFCM Profession V1.0). Transmission electron microscopy (TEM) was used to analyze exosomes. Exosomes were placed on a copper grid with excess exosomes removed, leaving a thin coat, and then wetted with 2% uranyl acetate in water. Grids were then dried overnight and TEM was performed.

Interactions between exosomes and injured tenocytes were assayed as follows: exosomes were stained using a PKH67 green fluorescent labeling kit (Sigma-Aldrich, St Louis, USA) according to manufacturer’s protocols and co-cultured with injured tenocytes for 24 h. Exosome endocytosis by injured tenocytes was assayed using a confocal microscope (Olympus FV1200, Tokyo, Japan).

### Study groups

Treatments were divided into five groups: *group 1*, injured tenocytes were treated with different concentrations of hucMSC exosomes (50, 100, and 200 μg/mL) for 48 h. *Group 2*, injured tenocytes were treated with 100 μg/mL hucMSC exosomes and the RhoA inhibitor CCG-1423 (1 μM; Selleck, Houston, USA) for 48 h. *Group 3*, injured tenocytes were treated with 100 μg/mL hucMSC exosomes and transfected with the ARHGAP5 overexpression vector for 48 h. *Group 4*, injured tenocytes were treated with 100 μg/mL hucMSC exosomes and transfected with the miR-27b-3p mimic, inhibitor, or NC for 48 h. *Group 5*, injured tenocytes were transfected with ARHGAP5 siRNA and treated with CCG-1423 (1 μM) for 48 h.

### Cell Counting Kit-8 assay

Following treatments, injured tenocytes (5×10 ^3^ cells/well) were plated in 96-well plates and cultured for 0, 12, 24, and 48 h. Then, 10 μL of Cell Counting Kit (CCK-8) solution (Signalway Antibody, College Park, USA) was added, and cells were incubated at 37°C in 5% CO _2_ for 1 h, after which the absorbance at 450 nm was determined.

### Transwell assay

Transwell assays were performed to measure the ability of injured tenocytes to pass through filters. After treatments, injured tenocytes were grown for 24 h in serum-free medium. Cells were trypsinized and seeded in Matrigel-coated (BD Biosciences, Bedford, USA) 24-well Transwell chambers (Costar, San Diego, USA). Then, 300 μL cell suspension was added to each insert and 700 μL DMEM plus 10% FBS was added to the lower chamber. After 48 h incubation at 37°C, invaded cells were fixed in 4% formaldehyde (Jinsan Chemical Reagent Co. Ltd., Chengdu, China) for 10 min and then stained in 0.5% crystal violet (Aladdin Chemical Reagent Co., Ltd., Shanghai, China) for 30 min. Cells were analyzed under a microscope at 200× magnification. Five randomly selected fields were counted for each chamber.

### Luciferase reporter assay

The ARHGAP5 3′-untranslated region (UTR) carrying a putative miR-27b-3p binding site was cloned into the pGL3 vector (Promega, Madison, USA). Using Lipofectamine 2000, injured tenocytes were transfected with miR-27b-3p mimic, inhibitor, or NC, pGL3-ARHGAP5-WT (WT) or pGL3-ARHGAP5-Mut plasmid (MUT), along with the pRL-TK vector (Promega) expressing Renilla luciferase. Cells were incubated for 48 h and luciferase reporter activity assays were performed. Renilla luciferase activity was used to normalize the relative luciferase activity.

### qRT-PCR

Total RNA was extracted using TRIzol reagent (Life Technologies, Waltham, USA). A PrimeScript kit (TaKaRa, Dalian, China) was used to synthesize cDNA following manufacturer’s instructions. qRT-PCR was performed using SYBR green PCR master mix (Applied Biosystems, Foster City, USA) on an ABI 9700 real-time PCR system (Applied Biosystems). To analyze miRNA expression, stem-loop real-time RT-PCR was conducted. Briefly, RNAs were converted to cDNAs using a cDNA synthesis kit (Thermo Fisher Scientific, Waltham, USA) and RT-PCR was performed using Maxima SYBR Green qPCR Master Mixes (Thermo Fisher Scientific) as per manufacturer’s instructions. The primers used for PCR are listed in **Table 1**. *GAPDH* and *U6* were used as internal references for mRNA and miRNA, respectively. The fold-changes of mRNA and miRNA levels were determined using the 2 ^−ΔΔCT^ method.

**Table:** **Table1** Sequences of primers used in this study

Gene	Primer sequence
*RhoA*	Forward: 5′-TTAGTCCACGGTCTGGTCTTC-3′ Reverse: 5′-CAGGCTCCATCACCAACAATC-3′
*ARHGAP5*	Forward: 5′-GGCGGATTCCATTTGACC-3′ Reverse: 5′-TTCTCGCTGATGCCTACC-3′
*ARHGAP6*	Forward: 5′-GAATTTGACCGTGGGATTG-3′ Reverse: 5′-CAGGGAGGTAGAAGGTATATG-3′
*ARHGAP10*	Forward: 5′-CGCCGCCCGATACTACATTC-3′ Reverse: 5′-GGATGCCCAGTCTCCAAAGC-3′
*ARHGAP18*	Forward: 5′-GGAATGCGAATACCCTTG-3′ Reverse: 5’-GTTGCTGCTTCTTGGTTG-3′
*ARHGAP21*	Forward: 5′-AGGAGGAAAGCACAGTAGAC-3’ Reverse: 5′-GCAAAGATGGAGGACACAAG-3′
*ARHGAP28*	Forward: 5′-CAGGAGAGGAGGAGAGTATG-3′ Reverse: 5′-GGTTGCCACAGGTATCAC-3′
*ARHGAP30*	Forward: 5′-TGAAAGCCAAAGGTAGAG-3′ Reverse: 5′-AGGGTCCAAGAACATAAC-3′
*ARHGAP35*	Forward: 5′-AGTATGAGTGGCTGGTGAGTC-3′ Reverse: 5′-CGGTGGATGTGCTGTAGAAAC-3′
*GAPDH*	Forward: 5′-AATCCCATCACCATCTTC-3′ Reverse: 5′-AGGCTGTTGTCATACTTC-3′
miR-27b-3p	Forward: 5′-GCGCGTTCACAGTGGCTAAG-3′ Reverse: 5′-AGTGCAGGGTCCGAGGTATT-3′
*U6*	Forward: 5′-CTCGCTTCGGCAGCACA-3′ Reverse: 5′-AACGCTTCACGAATTTGCGT-3′

### Western blot analysis

Protein lysates were prepared using RIPA lysis buffer containing a protease inhibitor cocktail (Sigma-Aldrich). Proteins were separated by sodium dodecyl sulfate-polyacrylamide electrophoresis, transferred to nitrocellulose membranes (Millipore), and blocked in 5% skim milk. Membranes were incubated with primary antibodies against ARHGAP5 (Biorbyt, St Louis, USA), ARHGAP28 (Invitrogen), ARHGAP18 (Abcam, Cambridge, USA), RhoA (Abcam), CD9 (Abcam), TSG101 (Abcam), ALIX (Abcam), Calnexin (Santa Cruz Biotechnology, Santa Cruz, USA) and GAPDH (Cell Signaling Technology, Danvers, USA), followed by incubation with horse radish peroxidase (HRP)-conjugated secondary antibodies (Beyotime, Shanghai, China). Signals were detected using an enhanced chemiluminescence system (Bio-Rad, Richmond, USA).

### Active small GTPase pull down assays

After treatments, injured tenocytes were subjected to RhoA-GTP pull down assays using GST-RBD (Pierce, Rockford, USA). RhoA-GTP levels in pull down lysates and total RhoA protein in whole cell lysates were measured by western blot analysis using an anti-RhoA antibody (Abcam).

### Achilles tendon injury in rats

Six-week male Sprague Dawley rats obtained from the Shanghai Laboratory Animal Center, Chinese Academy of Science (Shanghai, China) were randomly divided into three groups: control ( *n*=6), model ( *n*=6), and hucMSC exosomes treatment ( *n*=6). Rats in the model and exosome treatment groups were anesthetized with 10% chloral hydrate (Merck, San Diego, USA). After the left hind limb was shaved and disinfected, the Achilles tendon was exposed. The superficial Achilles tendon was removed, and a cut was made in the middle of the deep Achilles tendon, and then tendon repair was performed by Kessler suture (6–0 prolene suture; Ethicon, Edinburgh, UK). The rats in exosomes treatment group also received a single subcutaneous injection of hucMSC exosomes (100 μg) dissolved in PBS (50 μL). At 7 days post-surgery, animals were sacrificed and histological and immunohistochemical assessments were performed. All animal experiments were conducted according to the National Institutes of Health Guide for the Care and Use of Laboratory Animals. Experimental protocols were approved by the Animal Care Committee of Shanghai East Hospital. All efforts were made to minimize animal suffering.

### Histological assessment

Rats were humanely euthanized with an overdose of chloral hydrate. The Achilles tendons were harvested and fixed in 10% formalin, dehydrated, and embedded in paraffin. Tissue sections (4 μm thick) were prepared and stained with hematoxylin and eosin (H&E). Digital images were captured at 200× magnification using an Olympus BX51 microscope equipped with an Olympus DP71 charge-coupled device camera (Olympus). Image analyses were performed by blinded operators. Cellularity parameters were used to evaluate pathological changes during tendon injury: 0=no cell clusters, 1=sporadic small cell clusters primarily at boundaries, 2=several large cell clusters, and 3=high cellularity over the entire tendon tissue [ 23, 24]. Total histological score of a maximum of 3 points indicates a highly injured tendon.

### Immunohistochemical analysis

Immunohistochemical analyses of tendon sections were performed following standard protocols. Samples were stained using an anti-Ki67 primary antibody (Abcam) and incubated at 4°C overnight. Sections were washed and incubated with a HRP-conjugated secondary antibody (Cell Signaling Technology) for 30 min, and then examined under a Nikon Eclipse Ni-E microscope (Nikon, Melville, USA). Image J software (National Institutes of Health, Bethesda, USA) was used to calculate the percentage of positively stained cells in six different regions. The relative fold changes in other groups were determined using the percentage of cells staining positive for the antigen in the control normalized group.

### Statistical analysis

All experiments were conducted in triplicate and data were expressed as the mean±standard deviation (SD). All statistical analyses were performed using GraphPad Prism 8.0.2 (GraphPad Software, San Diego, USA). Group comparisons were performed using Student’s *t-*test or ANOVA followed by Tukey’s post-multiple test. A *P* value less than 0.05 indicated statistical significance.

## Results

### RhoA expression is downregulated in injured tendons and primary injured tenocytes

**Figure 1:**
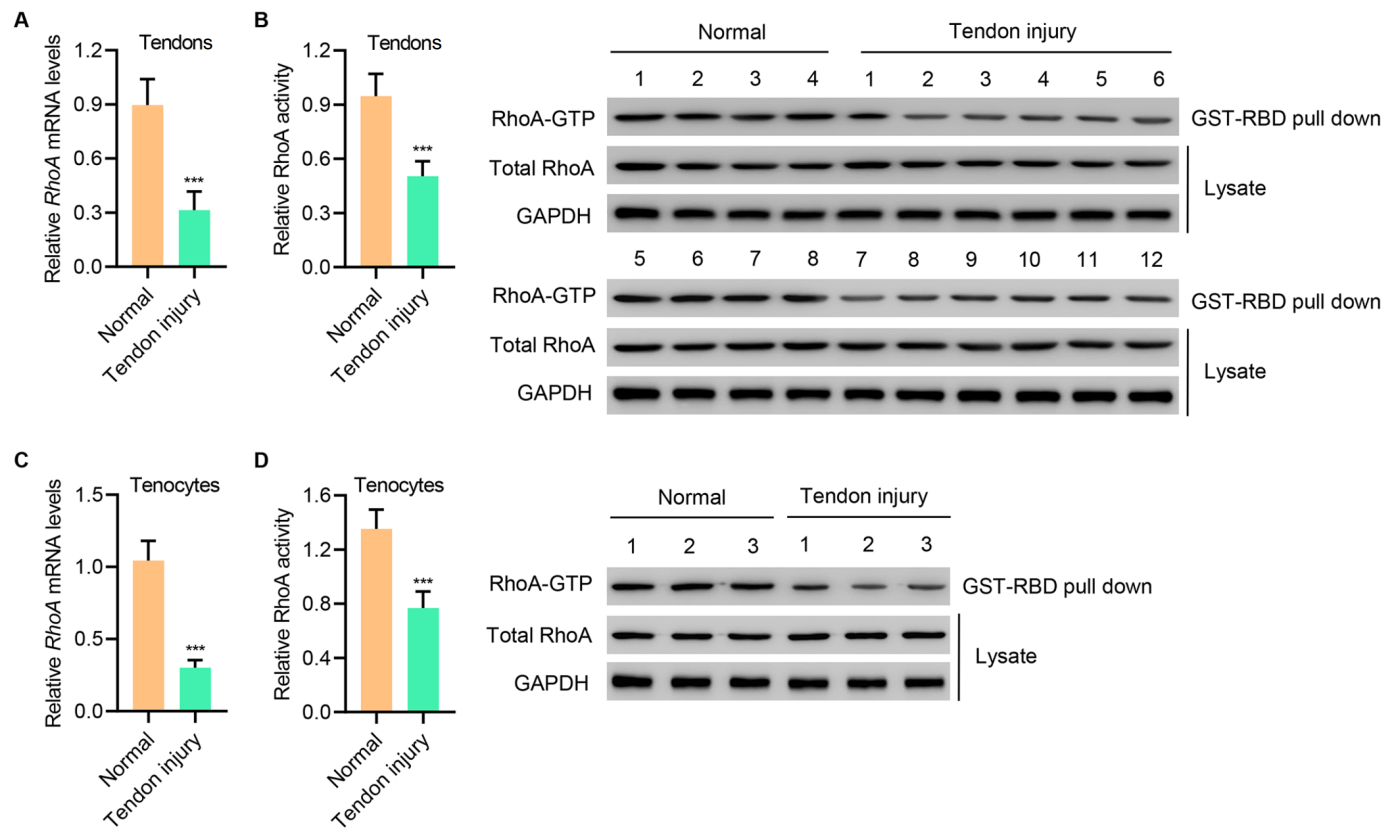
Figure1 **RhoA expression is downregulated in injured tendons and primary injured tenocytes**(A,B) RhoA mRNA expression and activity in tendons of patients with Achilles tendon tissue injury (n=12) or normal controls (n=8). (C,D) RhoA mRNA expression and activity in tenocytes of patients with Achilles tendon tissue injury (n=3) or normal controls (n=3). Data are expressed as the mean±SD (n=3, 8, or 12). ***P<0.001 compared with normal.

To determine the role of RhoA signaling in tendon healing and injury repair, we measured *RhoA* mRNA expression and activity in normal and injured tendon tissues. Interestingly, both indices were drastically reduced in injured tendon tissues when compared with normal tissues ( Figure 1A,B). Similar reductions were also observed in primary injured tenocytes when compared with normal tenocytes ( Figure 1C,D). Thus, RhoA signaling was reduced during tendon injury.

### HucMSC exosomes increase cell proliferation, invasion, and RhoA activity in primary injured tenocytes

**Figure 2:**
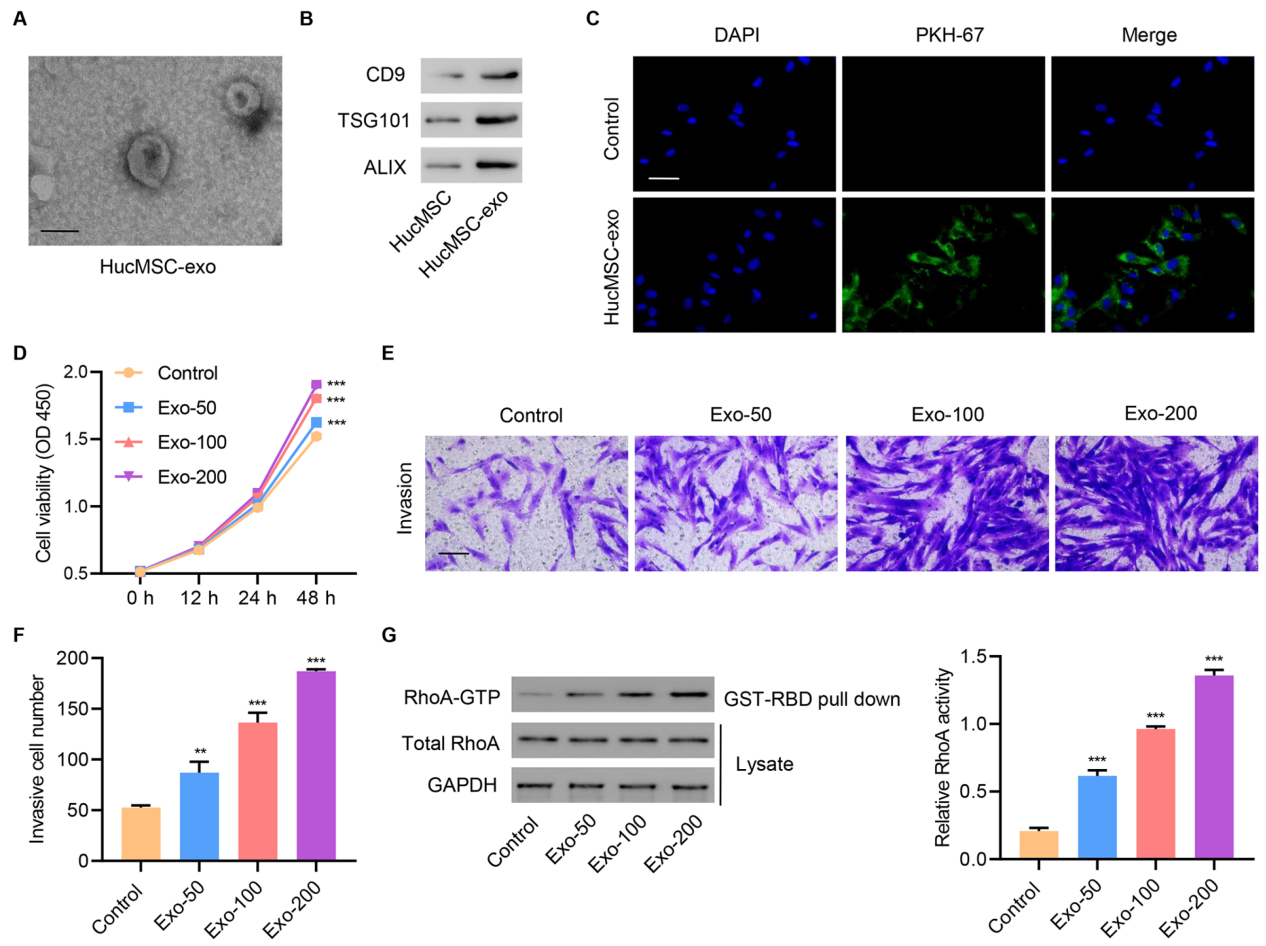
Figure2 **HucMSC exosomes increase cell proliferation, invasion, and RhoA activity in primary injured tenocytes**(A) A typical electron microscopy image of exosomes from hucMSCs. Scale bar=100 nm. (B) Representative western blots of CD9, TSG101, and ALIX proteins. (C) Laser scanning confocal microscopy was used to visualize exosome internalization by primary injured tenocytes. Scale bar=50 μm. Injured tenocytes were treated with different concentrations of hucMSC exosomes (50, 100, and 200 μg/mL), and (D) cell proliferation (E,F) invasion, and (G) RhoA activity were determined. Scale bar=100 μm. Data are expressed as the mean±SD (n=3). **P<0.01, ***P<0.001 compared with the control.

Before addressing the function of MSCs, we first determined whether the hucMSCs maintained the minimal characteristics of MSC. Isolated hucMSCs maintained their surface markers, including CD90 and CD105 positive expression, and CD11b and CD45 negative expression ( Supplementary Figure S1). To further investigate the role of hucMSC exosomes, we examined their effects on primary injured tenocytes. Purified exosomes from hucMSCs were small round vesicles and the size range of particles measured by NanoFCM was ~42 nm to ~197 nm, with an average size of ~69 nm ( Figure 2A and Supplementary Figure S2A). Successful exosome isolation was confirmed by western blot analysis using antibodies against different exosomal markers, including CD9, TSG101, and ALIX, and Calnexin as the non-exosomal marker ( Figure 2B and Supplementary Figure S2B). The internalization of hucMSC exosomes by primary injured tenocytes was further confirmed by confocal microscopy ( Figure 2C). To determine the role of hucMSC exosomes in tendon injury, different exosome concentrations were used to treat injured tenocytes, and then assessed the effects on cell proliferation, invasion, and RhoA activity. The results showed that increased exosome concentrations markedly enhanced cell proliferation ( Figure 2D) and promoted cell invasion ( Figure 2E,F). Moreover, exosomes increased RhoA activity in a dose-dependent manner ( Figure 2G), suggesting that exosomes regulated RhoA activity.

### RhoA signaling mediates the effects of hucMSC exosomes on cell proliferation and invasion

**Figure 3:**
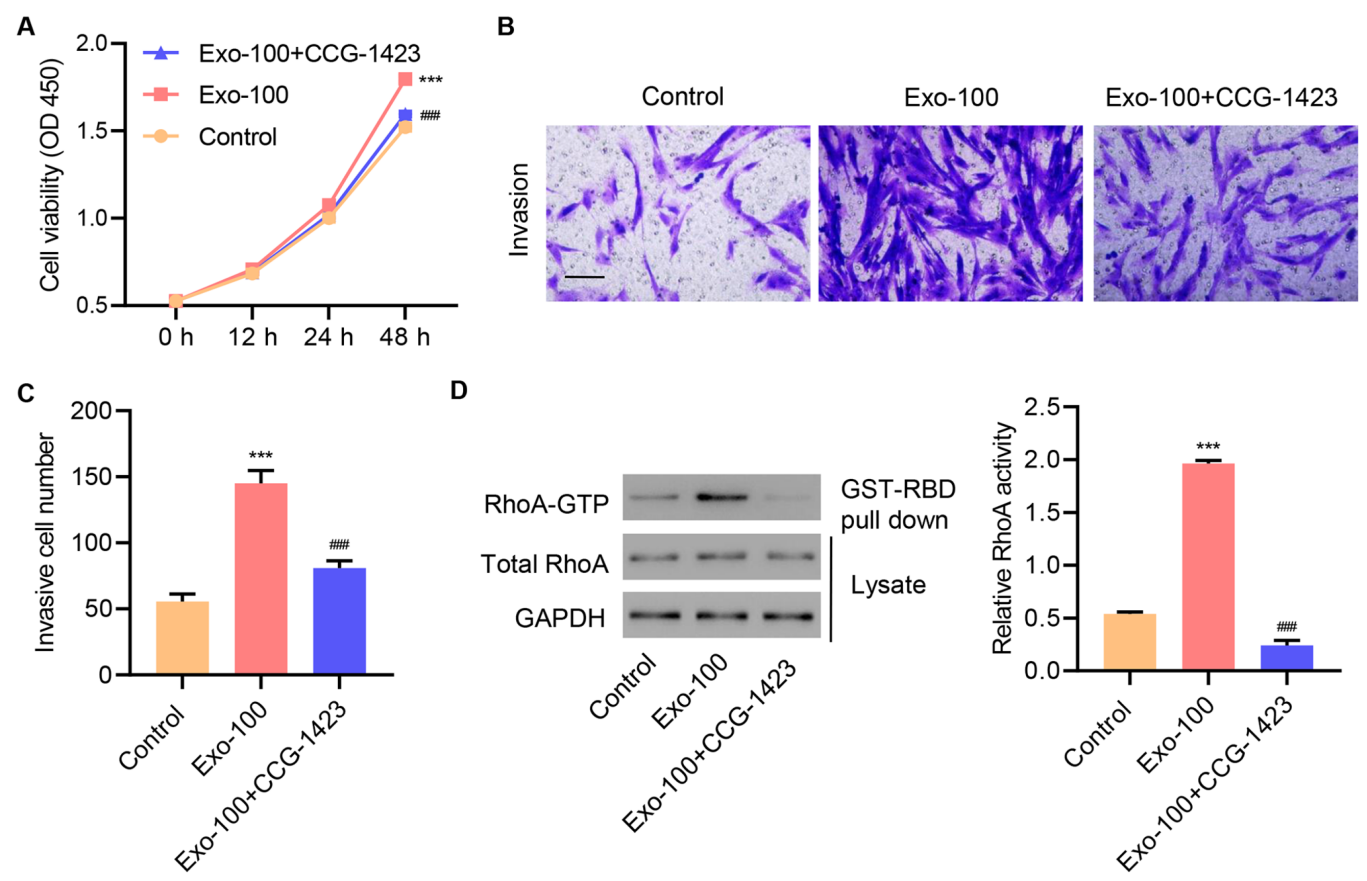
Figure3 **RhoA signaling mediates hucMSC exosome effects on cell proliferation and invasion**Injured tenocytes were treated with 100 μg/mL hucMSC exosomes and the RhoA inhibitor CCG-1423 (1 μM), and (A) cell proliferation, (B,C) invasion, and (D) RhoA activity were determined. Scale bar=100 μm. Data are expressed as the mean±SD (n=3). ***P<0.001 compared with the control. ###P<0.001 compared with Exo-100.

To determine whether RhoA signaling is an important mediator of hucMSC exosomes, injured tenocytes were treated with 100 μg/mL exosomes in the presence or absence of the RhoA inhibitor CCG-1423 for 1, 12, 24, and 48 h. As shown in Figure 3A, exosome treatment alone increased cell proliferation in a time-dependent manner, but CCG-1423 treatment suppressed exosome-induced increases in cell proliferation. Moreover, CCG-1423 treatment blocked exosome-induced cell invasion ( Figure 3B,C). Consistently, CCG-1423 treatment reduced GTP-bound RhoA protein levels and decreased RhoA activity ( Figure 3D). Collectively, these data suggested that RhoA signaling is a critical player in mediating the effects of hucMSC exosomes on cell proliferation and invasion.

### HucMSC exosomes increase RhoA activity in primary injured tenocytes by inhibiting ARHGAP5

**Figure 4:**
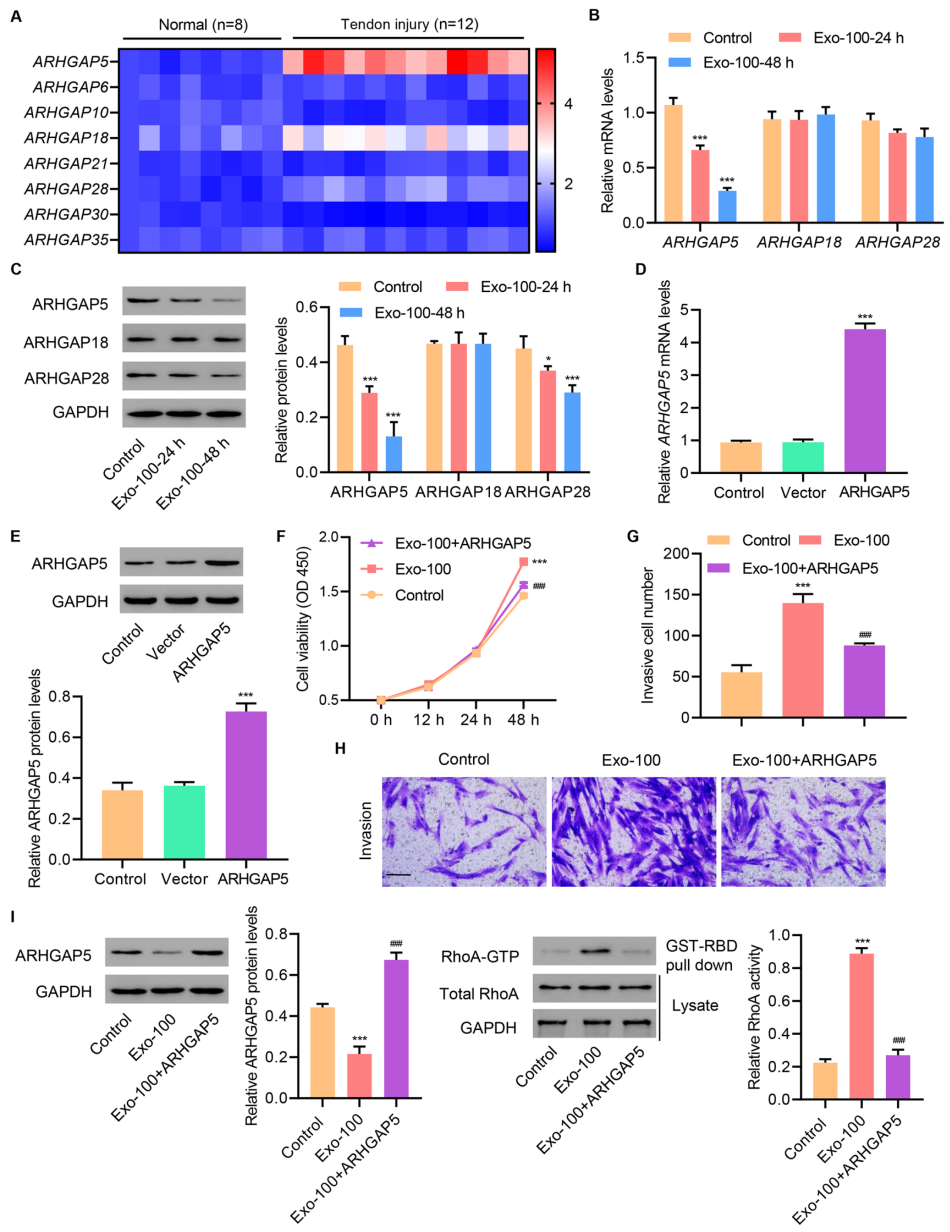
Figure4 **HucMSC exosomes increase RhoA activity in primary injured tenocytes by inhibiting ARHGAP5**(A) mRNA expressions of ARHGAP family members in tendons of patients with Achilles tendon tissue injury (n=12) or normal controls (n=8). (B,C) ARHGAP5, ARHGAP18, and ARHGAP28 expression in primary injured tenocytes treated with 100 μg/mL hucMSC exosomes for 24 and 48 h. (D,E) ARHGAP5 expression in primary injured tenocytes transfected with an ARHGAP5 overexpression vector. Injured tenocytes were treated with 100 μg/mL hucMSC exosomes and transfected with an ARHGAP5 overexpression vector, and (F) cell proliferation, (G,H) invasion, and (I) ARHGAP5 expression and RhoA activity were determined. Scale bar=100 μm. Data are expressed as the mean±SD (n=3). *P<0.05, ***P<0.001 compared with the control. ###P<0.001 compared with Exo-100.

We next investigated the mechanism through which exosomes increase RhoA activity in primary injured tenocytes. RhoA activity is modulated by GEFs and GAPs which function oppositely to one another: GEFs are positive regulators and GAPs are negative regulators [19]. We first checked the mRNA expression of different members of the Rho GAP protein family which act as negative regulators of RhoA activity. Specifically, we examined the mRNA expressions of *ARHGAP5*, *ARHGAP6*, *ARHGAP10*, *ARHGAP18*, *ARHGAP21*, *ARHGAP28*, *ARHGAP30*, and *ARHGAP35* in normal and injured tendon tissues. Interestingly, *ARHGAP5*, *ARHGAP18*, and *ARHGAP28* mRNA expression was dramatically increased in injured tendon tissues compared to those in normal tissues, whereas no significant changes were observed for the other *ARHGAP* members ( Figure 4A). Moreover, treatment of primary injured tenocytes with 100 μg/mL hucMSC exosomes for 24 and 48 h significantly downregulated ARHGAP5 at both transcriptional and protein levels, whereas no changes were observed in the other ARHGAPs, including ARHGAP18 and ARHGAP28 ( Figure 4B,C).

Next, we tested whether ARHGAP5 has a functional role in exosome-induced RhoA activity, and affects cell proliferation and invasion. Primary injured tenocytes were transfected with either ARHGAP5 overexpression vector or a vector control. As expected, ARHGAP5 overexpression elevated ARHGAP5 mRNA expression and protein levels when compared with the vector control ( Figure 4D,E). Functionally, ARHGAP5 overexpression blocked exosome-induced increases in cell proliferation ( Figure 4F) and invasion ( Figure 4G,H) of primary injured tenocytes, as well as in RhoA activity ( Figure 4I). These results suggested that hucMSC exosomes induced RhoA activation and increased cell proliferation and invasion by inhibiting ARHGAP5.

### ARHGAP5 silencing increases cell proliferation and invasion in primary injured tenocytes via RhoA activity stimulation

**Figure 5:**
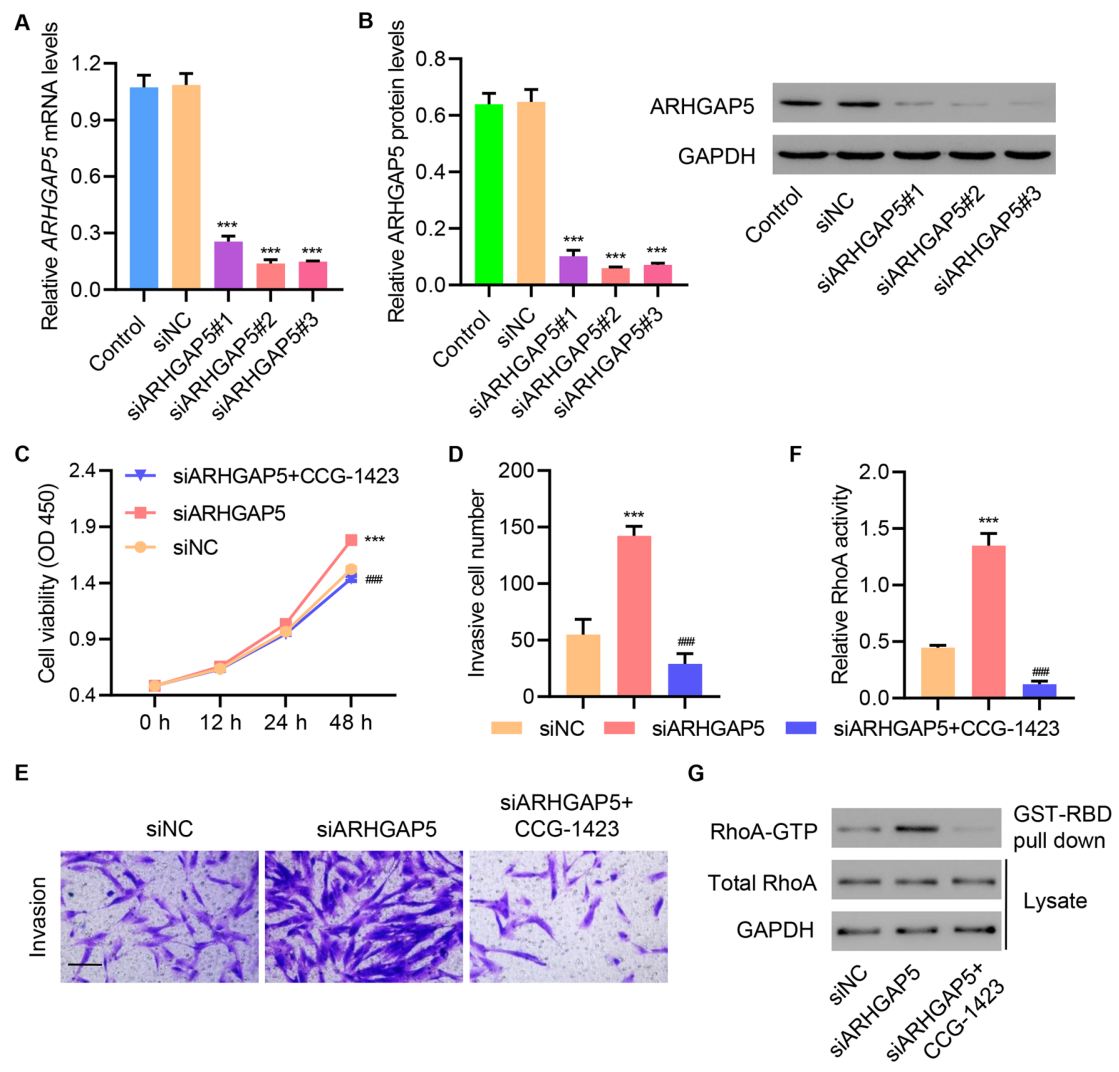
Figure5 **ARHGAP5 silencing increases cell proliferation and invasion in primary injured tenocytes by stimulating RhoA activity**(A,B) ARHGAP5 expression in primary injured tenocytes transfected with ARHGAP5 siRNA or siNC. Injured tenocytes were transfected with ARHGAP5 siRNA and treated with CCG-1423 (1 μM), and (C) cell proliferation, (D,E) invasion, and (F,G) RhoA activity were determined. Scale bar=100 μm. Data are expressed as the mean±SD (n=3). ***P<0.001 compared with siNC. ###P<0.001 compared with siARHGAP5.

To further establish the exosome-induced regulation of ARHGAP5 in the activation of RhoA and increase in cell proliferation and invasion, we silenced ARHGAP5 in primary injured tenocytes by transfecting siRNAs targeting ARHGAP5 or control siRNAs into the cells. As expected, ARHGAP5 siRNA transfection significantly downregulated ARHGAP5 mRNA and protein levels ( Figure 5A,B). Interestingly, transfection with siARHGAP5 dramatically increased cell proliferation, and this effect was abolished when the RhoA inhibitor CCG-1423 was supplemented to siARHGAP5-transfected cells ( Figure 5C). Similarly, siARHGAP5 transfection increased cell invasion, and this effect was abolished upon CCG-1423 supplementation ( Figure 5D,E). Moreover, siARHGAP5-transfected cells displayed increased RhoA activity as demonstrated by elevated protein levels of the GTP-bound RhoA, which were abolished by CCG-1423 treatment ( Figure 5F,G). Together, these data established that ARHGAP5 functions were regulated by RhoA signaling.

### HucMSC-derived exosomal miR-27b-3p increases cell proliferation, invasion, and RhoA activity in primary injured tenocytes

The potential of hucMSC exosomes in improving tendon adhesion has been investigated [11], and miR-27b-3p was identified to be highly expressed in hucMSC exosomes, which might mediate hucMSC function in the growth of vaginal epithelial cells [25]. To further investigate the mechanism of exosome-induced activation of RhoA and the regulation of cell proliferation and invasion, we examined the potential upstream ARHGAP5 regulators. As shown in Supplementary Figure S3, miR-27b-3p expression was found to be increased at different concentrations of hucMSC exosomes. These data suggested a potential mechanism through which hucMSC exosomes regulate ARGAP5 and subsequently affect RhoA activity, cell proliferation, and invasion.

**Figure 6:**
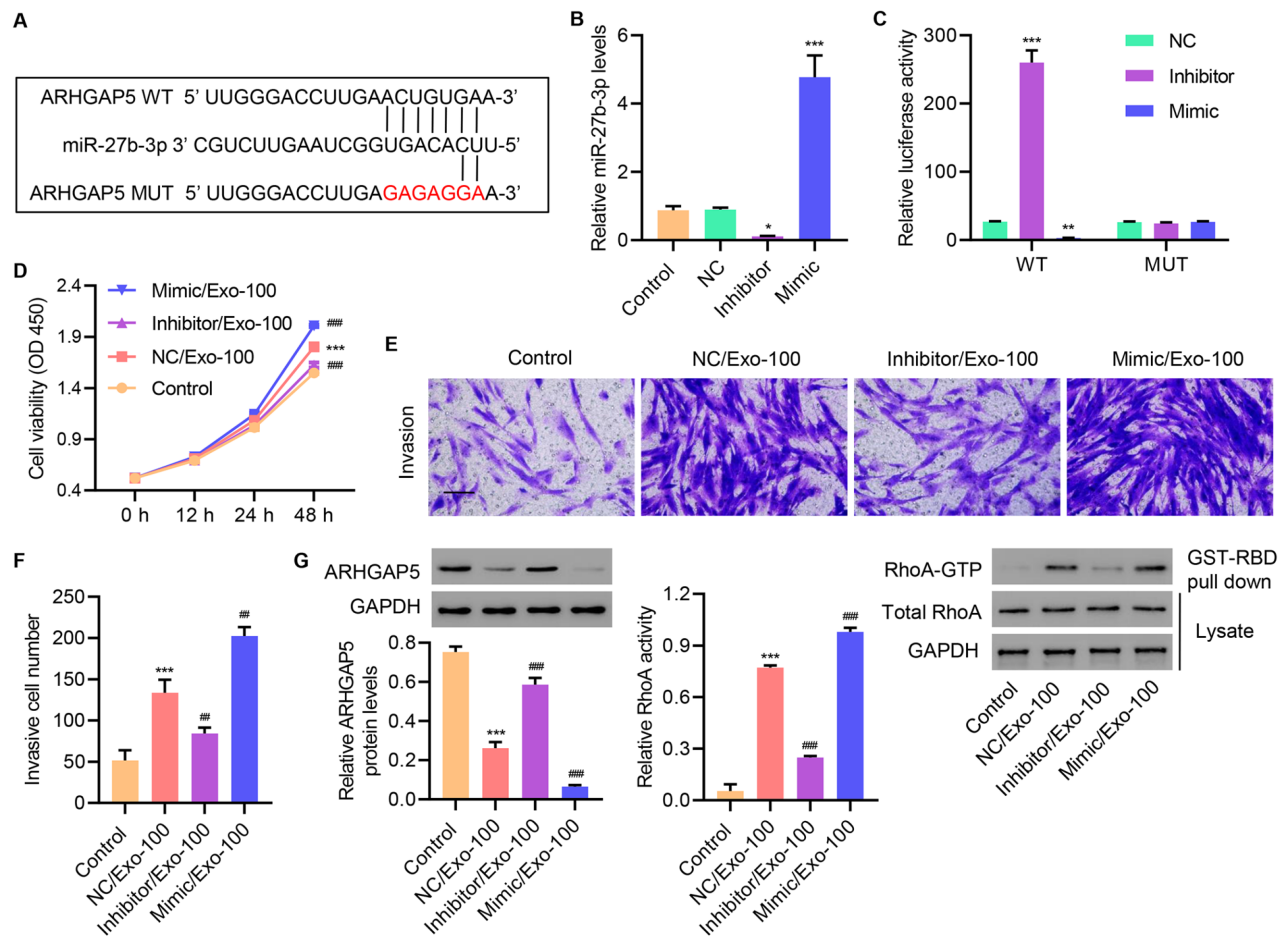
Figure6 **HucMSC-derived exosomal miR-27b-3p increases cell proliferation, invasion, and RhoA activity in primary injured tenocytes**(A) Binding sites of miR-27b-3p and ARHGAP5 predicted by miRDB database. (B) miR-27b-3p expression in primary injured tenocytes transfected with miR-27b-3p inhibitor, mimic or negative control (NC). (C) miR-27b-3p regulated ARHGAP5 3′UTR activity. Injured tenocytes were treated with 100 μg/mL hucMSC exosomes and transfected with miR-27b-3p mimic, inhibitor, or NC, and (D) cell proliferation, (E,F) invasion, and (G) ARHGAP5 expression and RhoA activity were determined. Scale bar=100 μm. Data are expressed as the mean±SD (n=3). *P<0.05, **P<0.01, ***P<0.001 compared with the control. ##P<0.01, ###P<0.001 compared with NC/Exo-100.

Interestingly, we identified a putative miR-27b-3p binding site in the 3′UTR of *ARHGAP5*( Figure 6A) by miRDB database. To manipulate miR-27b-3p expression in primary injured tenocytes, a microRNA inhibitor or mimic was used to upregulate or downregulate miR-27b-3p expression, respectively. As expected, miR-inhibitor transfection suppressed miR-27b-3p expression, whereas the miR-mimic elevated miR-27b-3p expression when compared with the NC miR and untransfected control ( Figure 6B). To test whether miR-27b-3p regulates the ARHGAP5 3′UTR, we mutated the predicted binding site of miR-27b-3p in the ARHGAP5 3′UTR and performed luciferase reporter assays to compare the regulation of wild-type (WT) versus mutant (MUT) ARHGAP 3′UTR. As shown in Figure 6C, transfection with the miR-inhibitor increased reporter activity in WT ARHGAP5 3′UTR-transfected cells, while miR-mimic transfection decreased reporter activity. Moreover, neither the miR-inhibitor nor the miR-mimic affected MUT ARHGAP5 3′UTR activity, suggesting that miR-27b-3p regulated the ARGHGAP5 3′UTR.

To investigate the role of miR-27b-3p on exosome-induced functions, we evaluated the effects of the miR-inhibitor and the miR-mimic on cell proliferation and invasion in primary injured tenocytes. As shown in Figure 6D, treatment with exosomes increased cell proliferation, and this effect was enhanced by the miR-mimic, but weakened by the miR-inhibitor. Similarly, exosome-induced increase in cell invasion was suppressed by the miR-inhibitor, but augmented by the miR-mimic ( Figure 6E,F). Consistently, exosome-induced decreases in ARHGAP5 expression and subsequent RhoA activation were reversed by the miR-inhibitor, but further enhanced by the miR-mimic, as revealed by western blot analysis ( Figure 6G). These results demonstrated the pivotal function of miR-27b-3p in mediating exosome-induced effects on ARHGAP5 expression, RhoA activity, and cell proliferation and invasion.

### HucMSC exosomes reduce tendon injury in an *in vivo* rat model

**Figure 7:**
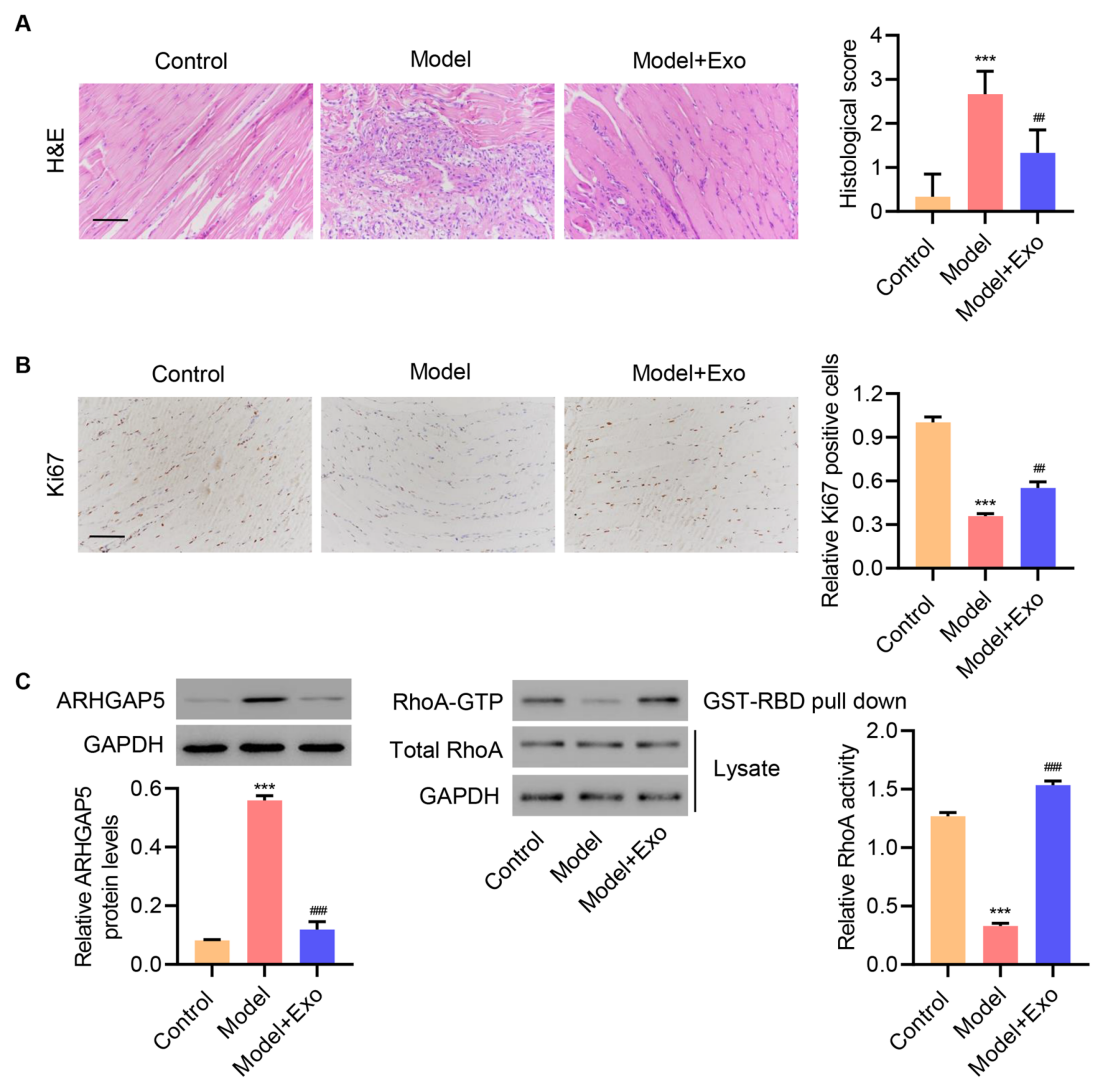
Figure7 **HucMSC exosomes inhibit tendon injury *in vivo*** (A) Exemplary histology of tendons using hematoxylin & eosin (H&E) staining (cellularity) and (B) immunohistological staining for Ki67, with (C) ARHGAP5 expression and RhoA activity determined in Achilles tendon tissues at 7 days post-surgery. Scale bar=100 μm. Data are expressed as the mean±SD (n=6). ***P<0.001 compared with the control. ##P<0.01, ###P<0.001 compared with the model.

Finally, to establish the physiological relevance of our findings *in vivo*, we investigated the effects of hucMSC exosomes on tendon injury in rats. Rats were subjected to Achilles tendon injury and treated with hucMSC exosomes or left untreated. After 7 days, H&E staining and immunohistochemistry were performed using anti-Ki67 antibody. H&E staining showed that normal Achilles tendons exhibited an integrated and continuous phenotype ( Figure 7A). Achilles tendons in the model group were disordered with hypercellularity, whereas repaired Achilles tendons appeared in the hucMSC exosome group, suggesting that exosome treatment reduced tendon injury in rats. Moreover, Ki67 staining indicated that exosome treatment rescued, at least in part, cell proliferation in injured rat tendon tissues ( Figure 7A,B). Consistent with the data from our *in vitro*primary injured tenocyte studies, exosome treatment reduced ARHGAP5 protein level and increased RhoA activity ( Figure 7C). These results supported a functional role for hucMSC-derived exosomes in reducing tendon injury by downregulating ARHGAP5 to activate RhoA signaling.

## Discussion

Tendon injuries are common clinical conditions and are an active research focus in the tissue engineering literature. Therapeutic approaches promoting tendon regeneration are of high clinical importance due to the limited healing capacity of tendon tissues. In recent years, there has been a general interest in cellular therapies that deliver adequate, regeneration-competent cell types to the injured tendon, to promote reconstruction and recover functionality. In this regard, MSC-based treatments have the potential to improve tendon healing and repair [11]. Moreover, MSC-derived exosomes help endogenous tendon stem and progenitor cells proliferate and migrate [26]. Consistent with these findings, we demonstrated a role for hucMSC-derived exosomes in reducing tendon injury by activating RhoA signaling. We further demonstrated mechanistic regulatory insights and showed that hucMSC exosomes induced RhoA activation by downregulating ARHGAP5, a negative regulator of RhoA. This regulation is mediated by miR-27b-3p which targets the ARHGAP5 3′UTR, resulting in ARHGAP5 suppression and RhoA activation. These findings offer exciting opportunities to develop potential therapeutic strategies for tendon injury repair and regeneration.

In recent years, it has become increasingly accepted that exosomes secreted by stem cells promote tendon injury healing [27]. In a study using a rabbit Achilles tendon injury model, bone marrow-derived MSCs affected early tendon healing [9]. Our *in vivo*data in an Achilles tendon injury rat model contextualized these findings and highlighted the role of hucMSC exosomes in tendon healing and regeneration. Our findings also supported the notion that exosomes released by MSCs improves tendon healing and regeneration.

The therapeutic induction of the miR-27b-3p/ARHGAP5/RhoA signaling was also exemplified by our study data. This pathway is a particularly attractive therapeutic target as the evidence suggests a role and involvement in tendon regeneration; Rho/Rock signaling was identified as a critical player in promoting tendon differentiation and contributing to tendon healing and repair [11]. Moreover, emerging studies have increasingly recognized the importance of microRNAs and exosomes in tissue regeneration. In a rat Achilles tendon adhesion model study, hucMSC exosomes delivered miR-21a-3p to inhibit tendon adhesion [28]. Similarly, our results indicated that hucMSC exosomes induced miR-27b-3p to enhance proliferation and invasion of primary injured tenocytes *in vitro*, and reduce tendon injury *in vivo*, supporting the therapeutic relevance of exosomes for tendon injury repair and healing. However, the effects of hucMSC exosomes directly transfected with miR-27b-3p mimics and antagomirs toward RhoA activity should be studied further. In addition, it will be interesting to determine whether the regulatory link from miR-27b-3p to ARHGAP5 to RhoA extends to other tissue injury systems.

In summary, our study highlighted the critical role of the miR-27b-3p/ARHGAP5/RhoA signaling network in tendon injury repair, and reinforced the findings of several studies on the therapeutic application of hucMSC-derived exosomes in tendon healing and regeneration.

## Supporting information

Supplementary_information

## Supplementary Data

Supplementary data is available at *Acta Biochimica et Biophysica Sinica* online.
