# Supplementary material for: Exosomes derived from human umbilical cord mesenchymal stem cells reduce tendon injuries via the miR-27b-3p/ARHGAP5/RhoA signaling pathway: Influences of hucMSC-exosomes in tendon injuries
Source: Acta Biochim Biophys Sin (Shanghai). 2022 Jan 26;54(2):232–42. doi: 10.3724/abbs.2021026 (PMC9909356; doi:10.3724/abbs.2021026)
Supplement: Supplementary_information [file Supplementary_information.doc]

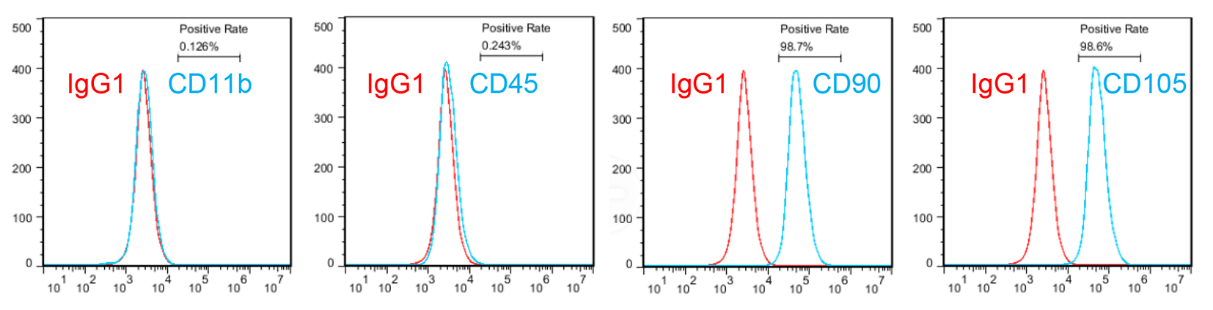


**Supplementary Figure S1. HucMSC characterization** HucMSC were stained positive with anti-CD90 and anti-CD105 antibodies, negative with anti-CD11b and anti-CD45 antibodies, which was identified by flow cytometry.


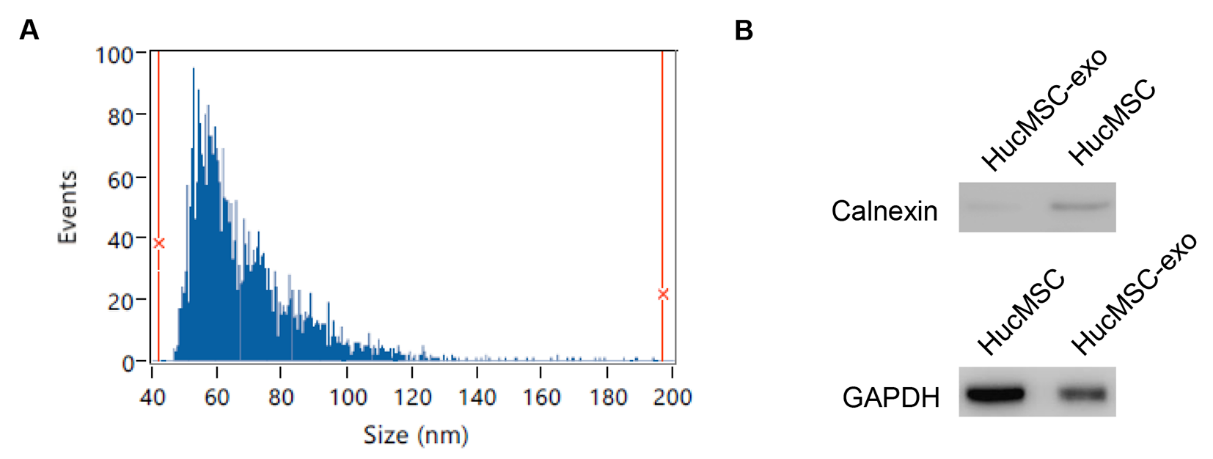


**Supplementary Figure S2.** **Characterization of exosomes from HucMSC** (A) Particle size distribution of exosomes from hucMSC measured by NanoFCM. (B) Western blots of Calnexin and GAPDH proteins.


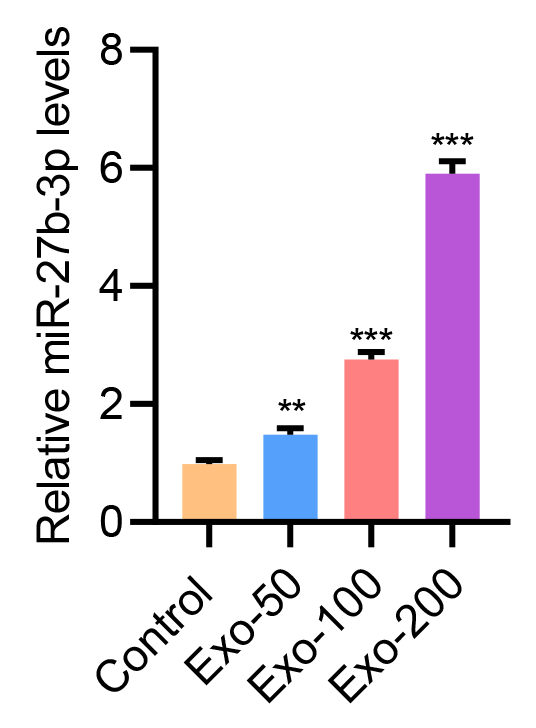


**Supplementary Figure S3.** **miR-27b-3p expression at different concentrations of hucMSC exosomes** Injured tenocytes were treated with different concentrations of hucMSC exosomes (50, 100, and 200 µg/mL), and miR-27b-3p expression was measured by quantitative RT-PCR. ***P*<0.01, ****P*<0.001 compared with control.
